# Supplementary material for: A mdg4 retrotransposon screen for X-linked female sterile alleles and its relationship with the transcription factor OVO
Source: G3 (Bethesda). 2026 Apr 17;16(6):jkag098. doi: 10.1093/g3journal/jkag098 (PMC13232525; doi:10.1093/g3journal/jkag098)
Supplement: jkag098_Supplementary_Data [file jkag098_supplementary_data.zip › Figure_S1_G3-2026-406610.pdf]

gypsy Female Sterile *ovo*^{D1} Reversion Test Crosses

Cross:	Number of Fertile Female Offspring:
y^{1} *v*^{1} *f*^{1} *mal*^{1} *lncRNA:flam*^{1} *su(f)*^{1}*/w*^{1118} *sov*^{ML150} X *ovo*^{D1} *v*^{24}	0/343 (0%)
FM7c, sn^{+} *P{lyB}lncRNA:flam*$^{py+P}$*/w*^{1118} *sov*^{ML150} X *ovo*^{D1} *v*^{24}	0/215 (0%)
y^{1} *v*^{1} *f*^{1} *mal*^{1} *lncRNA:flam*^{1} *su(f)*^{1} X *ovo*^{D1} *v*^{24}	2/222 (1.0%)
y^{1} *v*^{1} *f*^{1} *mal*^{1} *lncRNA:flam*^{1} *su(f)*^{1}*/FM7c, sn*$^{+}$ *P{lyB}lncRNA:flam*$^{py+P}$ X *ovo*^{D1} *v*^{24}	6/287 (2.1%)
