# Supplementary material for: A mdg4 retrotransposon screen for X-linked female sterile alleles and its relationship with the transcription factor OVO
Source: G3 (Bethesda). 2026 Apr 17;16(6):jkag098. doi: 10.1093/g3journal/jkag098 (PMC13232525; doi:10.1093/g3journal/jkag098)
Supplement: jkag098_Supplementary_Data [file jkag098_supplementary_data.zip › Supplemental_Material_Legends_G3-2026-406610.docx]

**Supplementary Legends**

**Figure S1: Cross scheme and reversion rates of *ovo^D1^*.**

**Table S1: Flybase ART Table.**

**Table S2: Meiotic mapping and complementation test results.** The interval that female sterility mapped to based on meiotic mapping using the phenotypic markers *y^1^*, *cv^1^*, *v^1^*, and *f^1^*. Black boxes indicate that the alleles were not complementation tested, green boxes denote alleles that complemented each other, red boxes denote alleles that failed to complement each other.

**Table S3: Characteristics of all non-*mdg4* dependent recessive female sterile alleles.**
